# Realization and Tests of Prototype Fluxgate Magnetic Sensors for the ITER Neutral Beam Injectors

**DOI:** 10.3390/s23031492

**Published:** 2023-01-29

**Authors:** Giuseppe Chitarin, Nicolò Marconato, Stefan Mayer

**Affiliations:** 1 Department of Management and Engineering, University of Padova, Strad. S. Nicola 3, 36100 Vicenza, Italy; 2 Consorzio RFX, CNR, ENEA, INFN, Università di Padova, Acciaierie Venete SpA, Corso Stati Uniti 4, 35127 Padova, Italy; 3 Department of Industrial Engineering, University of Padova, via Gradenigo 6a, 35131 Padova, Italy; 4 Stefan Mayer Instruments GmbH & Co. KG, Hans-Böckler-Str. 21 c, 46535 Dinslaken, Germany

**Keywords:** radiation-hard, magnetic sensor, vacuum compatibility, neutral beam injector

## Abstract

In the ITER neutral beam injectors (NBI), the presence of an external variable magnetic field generated by the ITER tokamak itself, could deflect the ion beam during acceleration and cause a loss of beam focusing. For this reason, the ion source, the accelerator and the neutralizer will be shielded from external magnetic field by means of a passive magnetic shield and a system of active correction and compensation coils (ACCC). The ACCC will operate in a feedback control loop and thus require the measurement of magnetic field inside the NBI vessel. Magnetic sensors for this application must be capable of measuring DC and slow variable magnetic fields, and be vacuum-compatible, radiation-hard and robust, since they will be subjected to neutron flux produced by fusion reactions in the tokamak and inaccessible for maintenance. This paper describes the realization and tests of fluxgate magnetic sensors prototypes specifically designed for this purpose before the installation in MITICA and ITER.

## 1. Introduction

**Figure 1:**
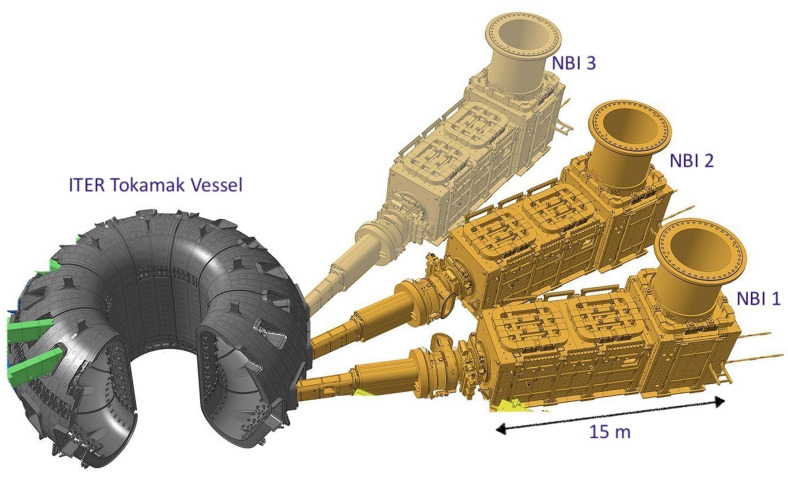
View showing the position of the 2 (+1) ITER NBIs (depicted in yellow) with respect to the ITER tokamak toroidal assembly. The volume of each NBI vacuum vessel, where the stray field shall be compensated, is about 15 × 2 × 2 m^3^; the active correction and compensation coils are located just above and below each NBI vessel (3 pairs).

ITER is a world-wide collaborative experiment for the demonstration of nuclear fusion as viable source of clean energy (www.iter.org). The experiments will be carried out in a toroidal device (tokamak) where high-temperature plasma required for fusion reactions will be confined using a strong magnetic field up to about 10 T. The ITER tokamak is presently under construction in Cadarache, in the south of France. Two heating neutral beam injectors (NBI) are foreseen in order to heat the plasma up to the required temperature [1]. A third NBI is presently considered as an option. MITICA (Megavolt ITER Injector and Concept Advancement) is the full-scale prototype NBI system for ITER [2,3] and is presently under construction in Padova, in the framework of a collaboration between the ITER International Organization (Cadarache, France), the Japan Domestic Agency of ITER (JADA) and Consorzio RFX. The injectors are designed to generate a bundle of 1280 H^–^ or D^–^ negative ion beamlets, with a negative-ion current up to 50 A, which are accelerated in a multistage electrostatic accelerator up to an energy of 1 MeV. After passing through a gas neutralizer, the accelerated particles shall constitute a single well-focused neutral beam of about 16.6 MW power, which will continue along a straight trajectory precisely directed towards the plasma in the ITER tokamak and transfer its energy to the magnetically confined plasma, as shown in Figure 1. However, in the NBI volume of interest, the ITER tokamak generates a fairly uniform stray magnetic field, always oriented along the vertical direction, ranging from a few hundred mT to several dozens of mT. This stray field can deflect the ion trajectories, hindering the acceleration and neutralization of the negative ion beamlets with a consequent loss of neutral beam focusing. In order to keep the magnetic field in the beam-pertinent volume below the admissible value of a fraction of mT, a combination of a two-layer passive magnetic shield (PMS) and active correction and compensation coils (ACCC), whose current will be driven by magnetic sensors in a feedback control loop, is foreseen for each ITER NBI. The ACCC are essentially three pairs of identical coils located just above and below each NBI vessel, similar to Helmholtz coils (the upper coils are visible in Figure 1).

### 1.1. General Requirements of Magnetic Sensors Suitable for ITER Neutral Beam Injectors

Due to the presence of the thick passive magnetic shielding tightly surrounding the NBI vacuum vessel, magnetic sensors for this application shall be installed in various locations inside the vacuum vessel near the beam source [4] and will be subjected to the neutron flux produced by fusion reactions in the tokamak and hardly accessible for maintenance. Thus, the sensors must be vacuum-compatible, immune to drift due to time integration and due to radiation effects on sensor materials, mechanically robust and suitable to remote-handling. Similar sensors are also foreseen for activating the interlock systems and for diagnostic purposes and therefore must comply with similar specifications.

Essentially, the measurement specifications of the magnetic sensor are the outcome of an integrated R&D and design endeavour to guarantee the operability of the ITER experimental device, whose components are, for the vast majority, first-of-a-kind equipment.

In fact, the absolute accuracy, bandwidth, time derivative and measurement range (standard and extended) of the magnetic sensor are the results of an overall physics and engineering development, involving the design of the passive and active magnetic shield (PMS and ACCC), as well as the physics of ion beam acceleration and neutralization, the accessibility of the NBI components, the neutron flux, the volume available for the shields and the power required for active field cancellation.

In the end, the PMS has been designed to reduce the stray magnetic field below 10 mT with a bandwidth of a few Hz, and a magnetic field sensor having a measurement range of 10 mT and a frequency bandwidth of about 10 Hz will be required for driving the feedback control of the ACCC.

### 1.2. Motivations for the Choice of Fluxgate Sensors, Separation of the Sensing Part from the Front-End Electronics

**Figure 2:**
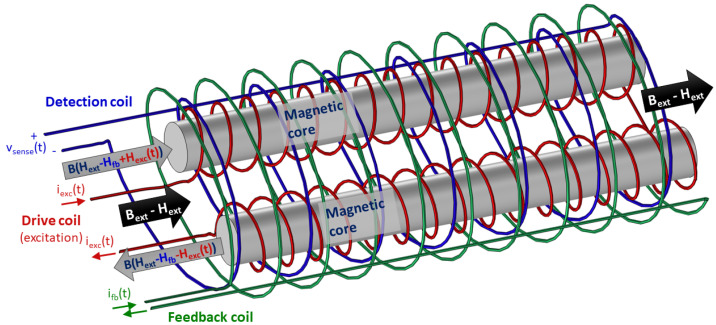
Conceptual scheme of a Vacquier-type fluxgate magnetic sensor (sensing part only). The sensor is constituted of a drive coil (excitation current) wounded in opposite direction around 2 magnetic cores and a detection coil (sense voltage) enveloping both. A feedback (or compensation) coil is added for cancelling the external field and thus increasing the output signal linearity and scale factor stability.

Semiconductor-based Hall sensors are not suitable to the ITER NBI application, because semiconductors would be heavily damaged by radiation from high-energy neutrons and gamma radiation after a short operation period [5]. Metallic Hall sensors can be used in these conditions, but their limited sensitivity [6] and complexity [7] would not allow their use for this specific application. MEMS sensors and magnetoresistive sensors have been considered several years ago [8,9,10] but have been abandoned due to their insufficient tolerance to radiation. Optical atomic magnetometers [11] can achieve sensitivity better than $pT/\sqrt{H}z$. However, such a high sensitivity is unnecessary for our application and their use would involve the installation of a glass bulb containing alkali gas and light sources, not suitable to be installed inside the NBI vessel. Pick-up coils (also called Mirnov coils) with a time integration of the output signal are not suitable, because the radiation-induced electromotive force (some microvolts, caused by neutrons hitting the coils conductors) would produce an unacceptable measurement drift during the long experimental pulses [12], but first and foremost because stationary or slow-varying magnetic fields must be sensed. It is possible to overcome these difficulties using a fluxgate-type magnetometer (see Figure 2), since its sensing part is simple, intrinsically robust and does not require delicate semiconductor components. Moreover, it does not require offset compensation, as it intrinsically provides an absolute measurement of the magnetic induction field, whereas other types of sensors measure the value relative to a reference [9,13]. Other reasons for selecting a fluxgate magnetometer for the present application are the following: A fluxgate magnetometer is intrinsically directional. Directional sensitivity is generally a key advantage for use in active magnetic field compensation systems where magnetic field components are controlled with compensation coils. Total field magnetometers such as proton precession magnetometers are excluded for such applications. Unlike optical magnetometers, a fluxgate sensor can be made from a very limited range of materials: soft magnetic cores, insulated copper wires and electrically insulating material for support structures are the only required materials. This makes fluxgate sensors an ideal choice for applications where only a limited selection of certified materials is available or allowed.

A fluxgate magnetometer can be manually assembled without the use of special tools, expensive machines or special techniques such as microfabrication. This makes it relatively easy to develop prototypes at low cost and in a short period of time.

However, commercial-production fluxgate sensors typically have a limited measurement range (typically below 1 mT) and are usually not radiation-hard, since the front-end electronics circuit (which includes semiconductor devices necessary for signal conditioning and processing) is enclosed together with the sensor in the same package.

The realization of a fluxgate sensor compatible with ITER NBI requirements has been demonstrated by means of a very basic prototype fluxgate sensor built at Consorzio RFX [14]. The experimental tests carried out on this prototype have shown that, with a suitable design of the magnetic core, the measurement range can reach about 10 mT, with a <10 μT accuracy and a bandwidth of 10 Hz.

### 1.3. Development Programme of ITER Fluxgate Magnetic Sensor

The development programme for the fluxgate magnetic sensors for ITER NBI and MITICA is expected to follow the following phases:

- **Phase 1:** Construction of two “engineered” fluxgate magnetic sensor prototypes. These sensors are designed to be a meaningful prototype of the fluxgate sensors which will be used in the MITICA neutral beam experiment and in the ITER NBI.
- **Phase 2:** Test and characterization of the “engineered” fluxgate magnetic sensor prototypes. Based on these prototype characterization tests, the technical specifications of the fluxgate magnetic sensors for MITICA neutral beam experiment will be finalized.
- **Phase 3:** A series of about 10 fluxgate magnetic sensors for MITICA will then be produced. These sensors will be installed and operated in the MITICA neutral beam experiment.
- **Phase 4:** Based on the operating experience with the fluxgate magnetic sensors for MITICA, the technical specifications for the production of the fluxgate sensors for ITER NBI will be finalized. These sensors will be installed and operated in the ITER NBI.

## 2. Description of the Design Requirements

It is important to keep in mind that the definition of design requirements and component specifications is itself the outcome of the research work, necessary for designing, integrating and realizing the ITER experimental device, whose components are, for the vast majority, first-of-a-kind equipment. The validation of these prototype sensors is a necessary step in this process. In each ITER NBI, 3 independently driven pairs of active correction and compensation coils (similar to Helmholtz coils) will be used for stray field cancellation along each beamline. For this reason, at least 3 vertically oriented fluxgate sensors are needed for each NBI.

The ACCC produce a magnetic field which is mainly vertical and of course can produce a limited field distortion in the adjacent volume. However, this distortion, which is also vertical in the volume of interest and relatively small, can be accounted for and compensated by the control system of the adjacent pair of coils.

A measurement range of ±2 mT is required for the “engineered” fluxgate sensor prototypes, with absolute accuracy of 20 μT (0.2 Gauss) within an expected operating temperature range of 20–40 °C. Temperature compensation circuits are included only if strictly necessary for achieving the required accuracy. Since the fluxgate sensors might also be used in higher-field regions of the ITER NBI, where strict measurement precision will not be required, the measurement range of the sensors is increased to ±10 mT, but no specific requirement on accuracy is defined in this case.

### 2.1. Functional Requirements: Measurement Accuracy, Range, Bandwidth and Operating Temperature

We summarize here the functional specifications of the “engineered” fluxgate magnetic sensor prototypes as mentioned in phase 1. Sensors will be installed in MITICA near the beam source, the neutralizer, the residual ion dump and the transmission line [1]. A summary of the requirements for these sensors is given in Table 1.

#### Vacuum and Neutron Radiation Requirements

Due to the position of the NBI injectors and to the fact that the NBI vacuum vessel will be directly connected via a duct to the ITER tokamak vessel, the expected neutron flux inside the NBI during the ITER D-T operation will be of the order of 10^13^ n/(m^2^ s) (considering 14 MeV neutrons) during the life of the ITER experiment (10^7^ s), corresponding to a neutron fluence of 10^20^ n/m^2^. This expected neutron fluence is approximately equivalent to a total dose of about 10^6^ Gray (or 10^8^ rad). These levels of neutron flux and fluence would seriously damage electronics components based on silicon semiconductors but are tolerable with acceptable damage by metals such as copper, steel and also by polyimide insulating materials such as Kapton® and Vespel SP-1®. The clear advantage of the fluxgate sensor is that the “sensing part” can be kept separated from the front-end electronics. The sensing (active) part is only constituted of ferromagnetic cores with coils made of insulated conductors, which also consist of radiation-resistant material such as mineral-insulated cables (MIC) and does not require any semiconductor-based component.

### 2.2. Sensor Description

**Figure 3:**
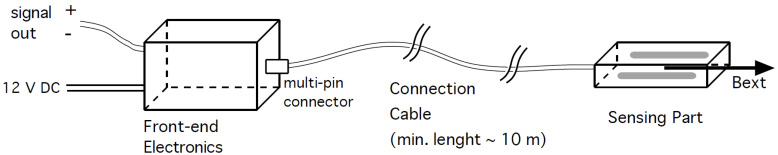
Main components of the “engineered” fluxgate magnetic sensor prototype.

The “engineered” fluxgate sensor prototypes are intended to be, as far as possible, identical to the sensors which will be used in the MITICA neutral beam experiment and in the ITER NBI (phases 3 and 4). For this reason, the fluxgate magnetic sensor prototypes are constituted of the following parts as shown in Figure 3:

- sensing part, consisting of ferromagnetic cores, excitation coils, a sensing coil and a feedback coil, designed to measure the magnetic field component B_ext_ along a well-defined “axial” direction. This part was designed so that it could be installed in the MITICA and ITER NBI vacuum vessel and is radiation-hard and vacuum-compatible, as described in the paragraph on sensor materials here below.
- Front-end electronics, consisting of the circuitry necessary for driving the excitation coils, for processing the sensing coil signal and driving the feedback coil, so as to produce an output voltage directly proportional to the magnetic field. This part will be installed outside the NBI vessel, will not be subjected to radiation and can be designed and built using standard industrial electronics technology and components.
- Connection cable, consisting of a bundle of copper cables connecting the sensing part to the front-end electronics. For the “engineered” fluxgate magnetic sensor prototypes, a vacuum-compatible cable having a minimum length of 10 m was considered sufficient in order to position the front-end electronics outside of the MITICA vessel. In the case of the ITER NBI, the cable length shall be of the order of 100 m, so as to position the front-end electronics outside of the bioshield.

**Figure 4:**
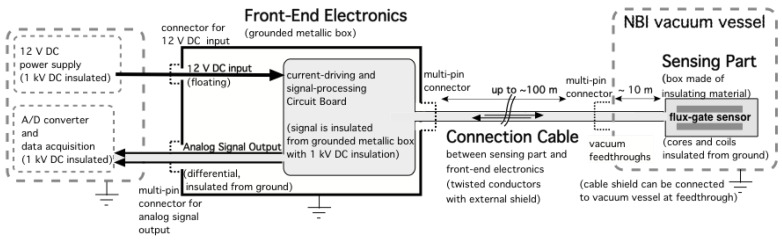
Signal paths and grounding scheme of the “engineered” fluxgate magnetic sensor prototype.

A conceptual scheme of the system from the probe to the data acquisition, showing also the grounding of the different parts, is shown in Figure 4.

### 2.3. Materials and Radiation Hardness

As indicated in the previous paragraph, the materials of the sensing part and the connection cable of the fluxgate magnetic sensors shall withstand a considerable neutron flux and fluence. Adhesives, solders and usual insulating materials are not allowed. The neutron flux and total fluence in the MITICA experiment will be about 100 times lower than in the ITER NBI and thus the “engineered” fluxgate magnetic sensor prototypes (phase 1) cannot be tested under a significant level of radiation. For this reason, radiation tests could be performed in parallel with phases 2 and 3, before production and installation of the ITER NBI magnetic sensors. All materials constituting the sensing part and the connection cables of the fluxgate sensor prototypes are also vacuum-compatible.

### 2.4. Sensing Part

The sensing part consists of ferromagnetic cores, excitation coils and a sensing coil, according to the scheme shown in Figure 2. A feedback compensation coil is also included for increasing the output signal linearity and stability. All components of the sensing part are enclosed in a containing box, made of nonmagnetic insulating material, machined to a tight tolerance, so as to guarantee accurate geometrical positioning of the core and coils and thus a precise orientation of the magnetic field measurement. The sensing part was designed in order to be suitable both for MITICA and ITER NBI, and all its components, including insulated coil wires, are radiation-hard and vacuum-compatible. For the enclosure and the other insulating parts, polyimide materials are used, such as Kapton® and Vespel® SP-1, which are compatible with the ITER NBI vacuum and radiation environment (vacuum quality classification VQC 2) [15]. As an alternative, alumina insulation and mineral-insulated cables (MIC) could also be used, as they are compatible with ITER vacuum quality classification VQC 1 [16]. The magnetic cores are made of soft ferromagnetic material, such as ferrite ceramic. Organic coating (for insulation and protection of the ceramic core from oxidation) is avoided. The coil reels or bobbins can be made of nonmagnetic material, such as sintered alumina or Vespel^®^ SP-1. The coils are made of copper conductors, with radiation-resistant and vacuum-compatible insulation, such as dipped Kapton^®^ wires. In order to avoid radiation-induced electromotive forces, all conductors are made of the same material (copper), soldered conductor junctions are avoided in the coils and in the connection cables. If necessary, only clamped connections (with bolts) or crimped connections are to be adopted. Provision is made to remove all bolted clamps and to install crimped junctions instead. This would be useful during phase 2, in case one of the “engineered” fluxgate magnetic sensor prototypes is subjected to neutron radiation tests. The insulation of the sensing part is designed to withstand a 1 kV DC voltage between the internal conductors and the outer surface of the containing box, for a duration of 60 s.

### 2.5. Front-End Electronics

The front-end electronics consists of the circuitry necessary for driving the excitation coils and for conditioning and processing the output signal produced by the sensing coil, which in turn is the error signal of the internal feedback loop of the sensor. The feedback coil current necessary to cancel the external magnetic field inside the sensor head (or sensing part) is directly proportional to the external magnetic field [17]. The feedback coil current is generated by a linear current source whose input voltage is used as the magnetometer output signal. The front-end electronics will not be subjected to neutron and gamma radiation and can be designed and built using standard industrial electronics technology and components. This is because in the MITICA experiment and in the ITER NBI, the front-end electronics will be installed in a protected diagnostics room, located outside of the NBI vessel and bioshield. The front-end electronics is enclosed in an electrically conductive metallic box, which will be locally grounded as shown in Figure 4. The front-end electronics box includes a current-driving circuit and signal-processing circuit on an electronics board, which is connected to the sensing part via the connection cable. A feedback circuit for driving the feedback compensation coil necessary for increasing the output signal’s linearity and stability is also included in the front-end electronics box. A multipin panel connector (LEMO^®^) is used for easily connecting and disconnecting the connection cable to the front-end electronics. The output of the signal-processing electronics will be transmitted to an insulated A/D converter and data acquisition via another multipin connector. The current-driving and signal-processing board and the connection cable are galvanically insulated with respect to the metallic box. This insulation is designed to withstand a 1 kV DC voltage for a duration of 60 s with respect to the metallic box which contains the front-end electronics. Even though the NBI vacuum vessel and the fluxgate sensors will be normally at ground potential, breakdowns in the 1 MV vacuum insulation of the ion accelerator are considered likely during operation and can possibly induce common-mode voltage surges up to several hundred volts in the connections. For this reason, particular attention was paid to the immunity of the sensor to induced and applied overvoltages.

### 2.6. Connection Cable

The connection cable is a shielded bundle of twisted copper conductors connecting the sensing part to the front-end electronics. The shield conductor of the cable will be connected to ground at an intermediate point (vacuum feedthrough, if any) and to the signal conditioning and processing circuits. All conductors and their connections on the sensing part are provided with radiation-resistant and vacuum-compatible insulation (see paragraph on sensor materials). Since multiconductor cables made of dipped Kapton wires with external shield are not commercially available, the connection cable was made in-house by inserting twisted pairs of copper wire (with “dipped” Kapton insulation) inside a braided metallic sleeve. The connection cable is attached to vacuum feedthroughs for tests under vacuum (phase 2). In the MITICA Neutral Beam experiment and the ITER NBI (phases 3 and 4), a total cable length of about 100 m is foreseen. However, only the first section, from the sensing part to the vacuum feedthrough (about 10 m) shall be radiation-hard and vacuum-compatible. A solution for the connection between the vessel feedthroughs and the front-end electronics has to be developed later on, during phase 2.

## 3. Engineering, Implementation and Construction

**Figure 5:**
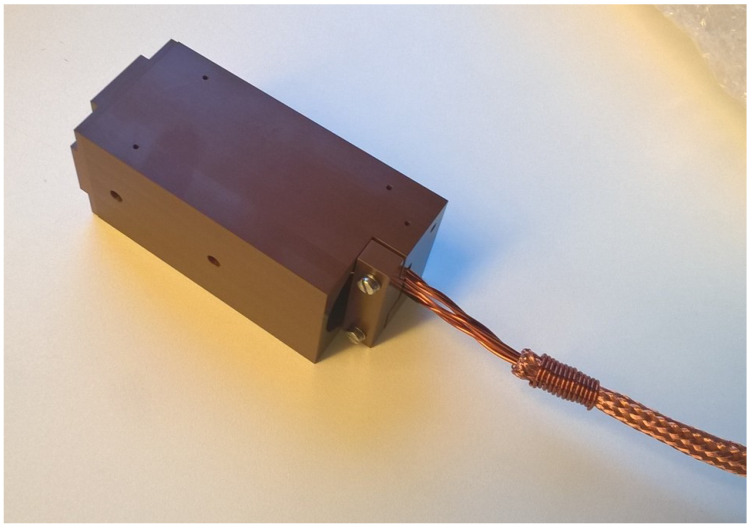
Picture of the assembled MITICA fluxgate sensing part.

**Figure 6:**
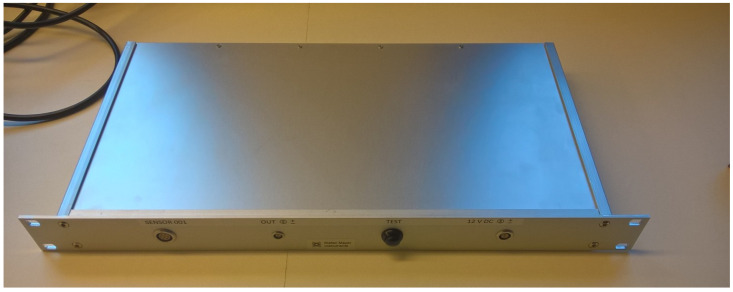
Picture of the enclosure of the front-end electronics box.

**Figure 7:**
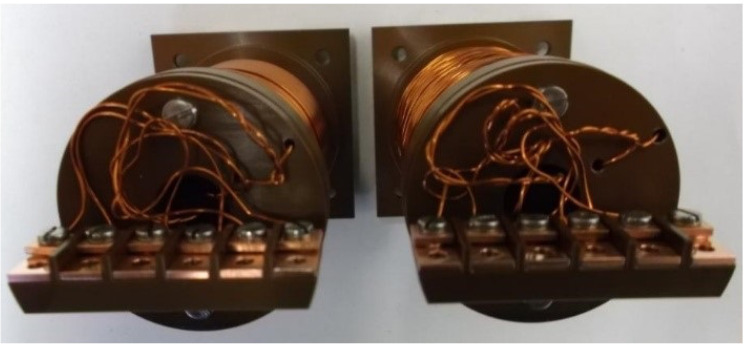
Picture of the coil windings around the peek reel, showing the system for the connection to the external cable.

Two fluxgate sensors, including sensing part, connection cable and front-end electronics necessary to drive the excitation coils and to acquire, condition and process the output signal from the detection coil were built based on the manufacturer’s experience of about 30 years (see Figure 5, Figure 6 and Figure 7).

### 3.1. General Design Ideas

The main challenge of the fluxgate sensor design was to obtain a fluxgate sensor with the unusual high measurement range of 10 mT from a limited selection of allowed materials while assuring operation in vacuum where convection cooling does not exist. The large measurement range required a comparable large excitation current amplitude as well as a large feedback magnetic field amplitude. On the other hand, the power consumption of the sensing part must be low enough to keep the operating temperature in vacuum within certain limits to avoid signal drift or even damage of the components. The idea was to keep the power consumption of the sensing part so low that self-heating in free air without mounting the sensing part to a heat sink was no more than a few degrees. It was assumed that the sensing part would be mounted to a heat-conducting surface in the final vacuum application so that missing convection could be at least partly compensated by additional conduction so that the temperature increase in vacuum would be no more than a few 10 degrees in the worst case. Accurate temperature measurements and heat flux estimates have not yet been carried out. The cores and coils were therefore designed no larger than necessary for easy assembly in order to keep the coil impedances low.

### 3.2. Construction of the Sensing Part

Two cylindrical rods of MnZn soft ceramics ferrite (Fair Rite Type78) were used as magnetic cores. The magnetic permeability of the ferrite material was considerably lower than that of high-permeability materials (e.g., cobalt alloy) which are commonly used for fluxgate sensors but turned out to be still high enough to achieve a signal resolution better than required. The core diameter was 2.0 mm and the length was 15 mm.

The cores were surrounded by cylindrical excitation coils, each with 92 turns of 0.14 mm thick Kapton insulated copper wire (Allectra 311-KAP-014) wound on bobbins made from Vespel SP-1. A single detection coil consisting of 378 turns of the same wire was wound on a Vespel bobbin with a 7.7 mm diameter. The length of the winding was 16 mm. The excitation coils were fixed in parallel cylindrical holes inside the bobbin to make a Vacquier type fluxgate sensor [9,18].

The excitation and detection coil assembly was inserted into a 45 mm long solenoid consisting of 2300 turns of 0.4 mm thick Kapton-insulated copper wire (Allectra 311-KAP-040) wound on a Vespel bobbin with a 13 mm inner diameter. This solenoid served as the feedback coil to compensate for up to 10 mT of external field.

The complete coil assembly was surrounded by a rectangular machined Vespel enclosure with 90 mm of length (in measurement direction) and 42 mm of width and height (see Figure 5). The parts were fixed with A4 stainless-steel screws. Venting holes were drilled into the parts at several places to enhance the vacuum compatibility. This enclosure was machined to a tight tolerance defined by the design requirements specified above, so as to guarantee accurate geometrical positioning of the cores and coils and thus a precise orientation of the magnetic field measurement, as resulted from the acceptance tests described in the following. The coil wires were connected to the connection cable via copper clamps fixed with A4 stainless-steel screws to a terminal block attached to the feedback-coil bobbin. Figure 7 shows a picture of the coil assembly and the terminal block for the connection to the external cable. A small Vespel block screwed to the enclosure served as cable strain relief clamp. The assembled sensing part is shown in Figure 5. All components of the sensing part and the connection cable were made from materials in compliance with the ITER vacuum handbook [15,16], except for the cable connector.

#### Dimensions and Tolerances

The overall size of the sensing part was chosen on the basis of the following considerations: Since the magnetic field inside the passive magnetic shield and the NBI vessel was expected to be fairly uniform on the scale of ∼0.1 m, the size of the box mainly resulted from the requirements for the sensor feedback coil made of dipped Kapton wires, that is, the coil had to be large enough to generate a homogeneous magnetic field of up to 10 mT over the volume of the magnetic cores while the self-heating was kept so low that any signal drift did not exceed the specifications. On the other hand, the size of the sensing part should not be larger than necessary to limit material costs and to ensure easy handling and installation. The choice of electrically insulating material allowed to be used inside the NBI vessel (primary vacuum of ITER tokamak) was limited to pure alumina and Vespel. The heat generated in the sensing part was so low that the selection of electrical insulating material with a high heat conductivity was not considered at this stage of the development (and would probably be difficult to find among the few allowed materials). The box containing the sensing part was a parallelepiped made of insulating material (Vespel SP-1), 90 × 42 × 42 mm in size. The box was machined with a tolerance better than 0.1 mm with respect to nominal dimensions. The box was designed in order to guarantee the geometrical alignment of the sensor cores with respect to the box. The orientation of the measured magnetic field component was orthogonal within 0.5 deg with respect to an external reference surface indicated on the containing box. The front-end electronics metallic box can be inserted in a standard 19-inch rack (see Figure 6).

### 3.3. Manufacturing of the Connection Cable

The insulation of all the internal circuits and connectors as well as the box of the sensing part were designed to withstand a voltage of 1 kV for 60 s.

### 3.4. Electronics Description

The front-end electronics will not be exposed to radiation and was designed and built using standard commercial through-hole mounted electronics components soldered to a standard FR4 double-sided printed circuit board.

**Figure 8:**
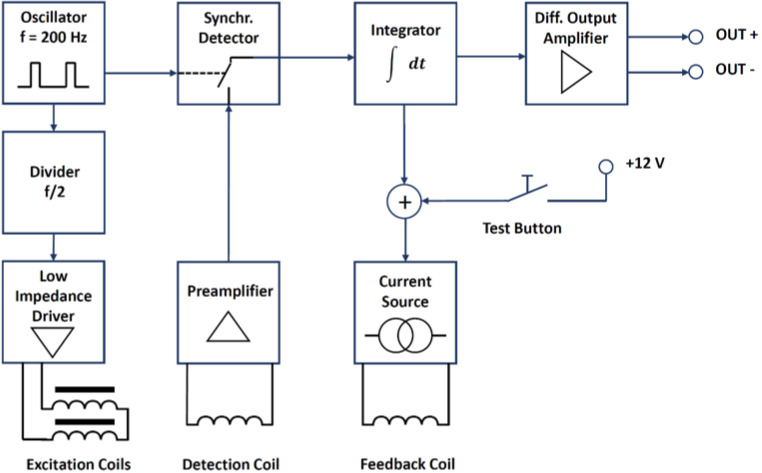
Conceptual scheme of the fluxgate magnetic sensor. The sensing part includes a feedback coil in order to achieve the required measurement range.

The electronics metallic box is a standard 19 aluminium rack-mounted enclosure which will be locally grounded as shown in Figure 3 and contains the coil driving, detecting and feedback electronics. Figure 8 shows a schematic block diagram of the different front-end electronics sections together with the excitation, detection and feedback coils.

The front-end electronics provides an analogue output signal proportional to the measured magnetic flux density component along the sensing axis with a range of ±10 V.

The system requires a power supply feeding a well-regulated DC voltage of 12 V ± 5%. The power input is protected against overvoltage and voltage reversal with a transient suppressor diode able to withstand 1 A of permanent forward current. The differential analogue output voltage is not floating with respect to the power supply and the signals can swing from the negative to the positive rail of the power supply voltage. The enclosure is insulated from all electrical signals. The sensor cable shield is connected to half the power voltage via a buffered voltage divider (virtual ground).

The fluxgate excitation frequency was chosen as low as possible to reduce the power consumption and heat produced in the sensing part. However, the excitation frequency must be considerably higher than the required measurement bandwidth of 10 Hz in order to guarantee feedback loop stability and a low excitation ripple on the output signal. The excitation frequency was therefore set to 100 Hz. External magnetic field with frequency near twice the excitation frequency or harmonics of twice the excitation frequency can result in beat frequencies seen on the fluxgate output signal. A magnetic field containing a considerable quantity of 200 Hz or even higher harmonics of the power line frequency of 50 Hz is very unlikely to occur in most lab environments. The main observed magnetic field frequencies are 50 Hz and only odd harmonics. However, it is easily possible to change the excitation frequency to different values in case problems with beat frequencies occur.

The excitation coils are connected in series and the excitation current is supplied by a low-impedance driver. A special drive circuit [19] involving a tank capacitor is used to generate short current pulses with a pulse width only limited by the excitation coil inductance (so-called ferro-resonance excitation mode FEM [20]). The low duty cycle helps to reduce the power consumption while driving the cores into saturation. The detection signal is rectified with a 2nd harmonic synchronous detector made from an analogue switch controlled by a signal with twice the excitation frequency and adjustable duty cycle. The rectified detection signal is integrated over time. The integrated signal controls a current source which drives the feedback coil. The integral feedback path is required to ensure complete compensation at DC as the integrator gain increases with decreasing frequency. A proportional or differential path as in a PID controller is omitted to avoid feedback of the second harmonic of the excitation frequency which otherwise would cause loop instability. The integrator therefore also serves as a low-pass filter for the feedback signal. Once the feedback loop is closed, the integrator output signal is proportional to the magnetic field to be measured. The feedback compensation loop ensures that the cores are always operated at the same working point near zero DC magnetic field, thus greatly improving the output signal linearity. The scale factor can be easily adjusted by adjusting the transfer ratio of the current source. The integrator output signal is fed to a differential output amplifier. The output signal of the differential amplifier is proportional to the feedback current required to keep the magnetic cores in zero field and it is therefore proportional to the external magnetic field. The single-ended integrator output signal is transformed into a differential signal to enhance the immunity against noise pick-up by the long connection cable.

A TEST button is foreseen to inject a DC current in the feedback coil producing a magnetic field of about 1.3 mT, which results in an output voltage of +1.3 V.

## 4. Tests

### 4.1. Factory Acceptance Tests

**Figure 9:**
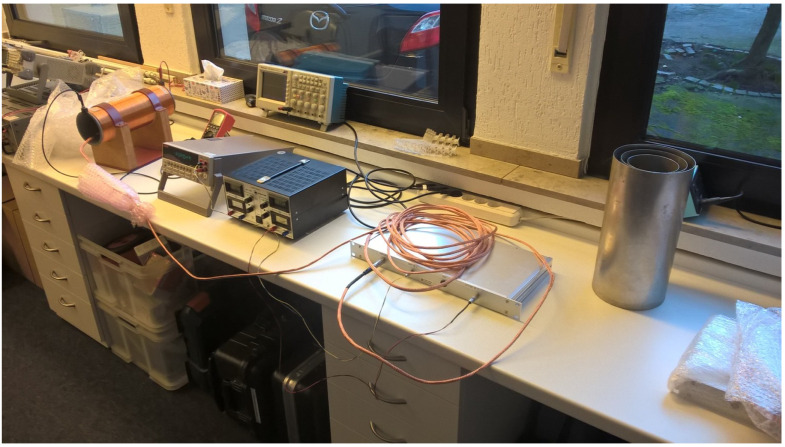
Acceptance test setup, the solenoid is visible on the left, the zero-gauss chamber is visible on the right.

**Figure 10:**
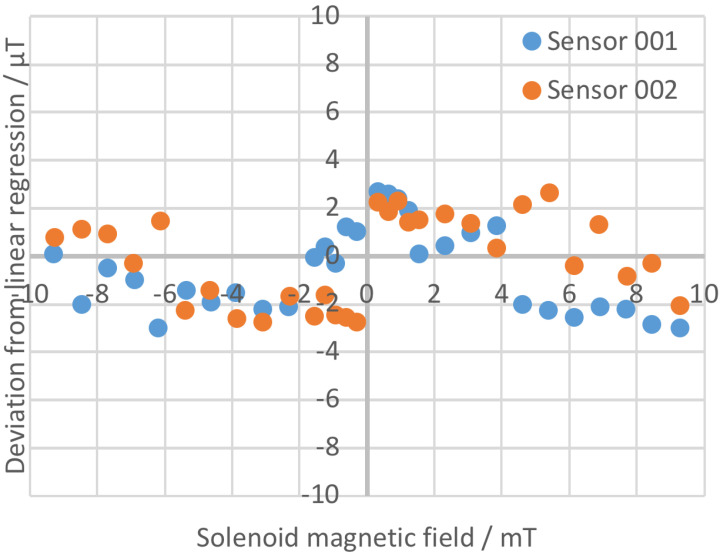
Linearity test results at the Stefan Mayer Instruments GmbH factory.

The following factory acceptance tests were carried out (Figure 9), in order to assess the technical specifications defined and summarized in Table 1:

1. Linearity and sensitivity test;
2. Offset error and noise test;
3. Bandwidth test;
4. Alignment and immunity test.

1. The linearity tests were carried out by placing the sensing part inside a calibrated solenoid with its axis oriented in the east–west direction. Different DC current values were applied and measured with a calibrated current meter (Keithley 2000), whereas the fluxgate sensor output signal was measured with a calibrated voltmeter. The results of the linearity tests are shown in Figure 10.
  The linearity tests demonstrated a deviation lower than 4 µT in a range of ±10 mT with a minimum absolute value of the magnetic field of about 0.3 mT. The scale factor of the output signals was set to 1 V/mT with an overall accuracy better than 0.5%. The measurement range (par. 1), absolute accuracy (par. 3) and analogue output voltage range of ±10 V (par. 4) were therefore satisfied.
2. The offset error and noise tests were carried out by placing the sensing part in a three-layer mumetal shield, shown in Figure 10. The tests showed an offset lower than 100 nT and a noise of about 30 nTpp in the range 0.1÷10 Hz, thus confirming that the requirement on sensitivity (par. 3, <0.01 mT) was fully accomplished.
3. For the bandwidth test carried out at 1 mT from 1 to 15 Hz, the calibrated solenoid used for the linearity test was connected to a Kepco BOP 20-20M four-quadrant power amplifier controlled by a frequency generator. Test results showed the capability of measuring an AC magnetic field with frequency up to 15 Hz with a response decrease of about −3 dB. The requirement on the measurement bandwidth (par. 8) was therefore confirmed.
  This tests also demonstrated the time derivative of the magnetic field requirement of 10 mT/s was satisfied (par. 7).
4. The alignment tests were carried out by placing the sensing part in Helmholtz coils with a 600 mm diameter. The coils produced a vertical magnetic field of 100 and 200 µT. The sensing part’s measuring axis was aligned horizontally pointing east. In order to correct for alignment errors of the Helmholtz coils, the test was repeated with the sensing part’s measuring axis pointing west and the results were averaged. The error in the alignment of sensor package with sensor reference surfaces was found to be lower than ±0.2° and therefore lower than required (par. 10 was satisfied). The test showed an immunity to transverse magnetic field of about −43 dB, which was better than the required value of −40 dB (par. 11 was accomplished).

### 4.2. Specific Tests at Consorzio RFX (Temperature Effect, Long Cable and High Voltage)

Further tests were carried out at Consorzio RFX in order to verify the remaining requirements. These are the parameters not highlighted in bold in Table 1, essentially the temperature stability and the voltage holding up to 1 kV, and the possibility to increase the distance between the sensing part and the front-end electronics using a 100 m long extension cable.

**Figure 11:**
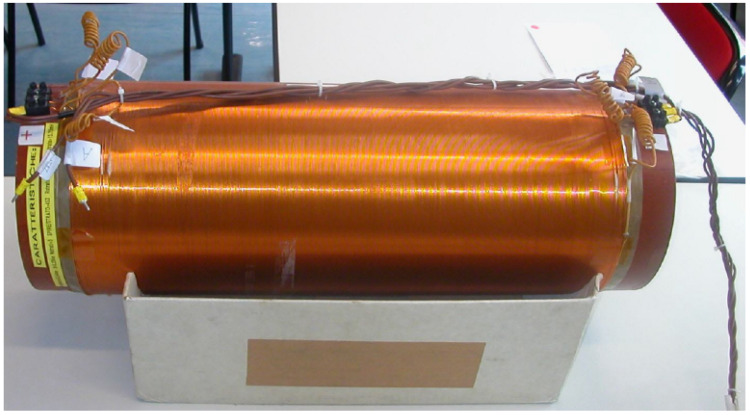
Solenoid adopted for the tests at Consorzio RFX.

In order to verify the effect of temperature and connection cable length, the linearity tests were repeated in different conditions. These were carried out again by placing the sensing part inside a solenoid with its axis oriented in east–west direction. The solenoid (Figure 11) was made up of three layers of 453 turns 545 mm long, with an inner radius of 221 mm.

**Figure 12:**
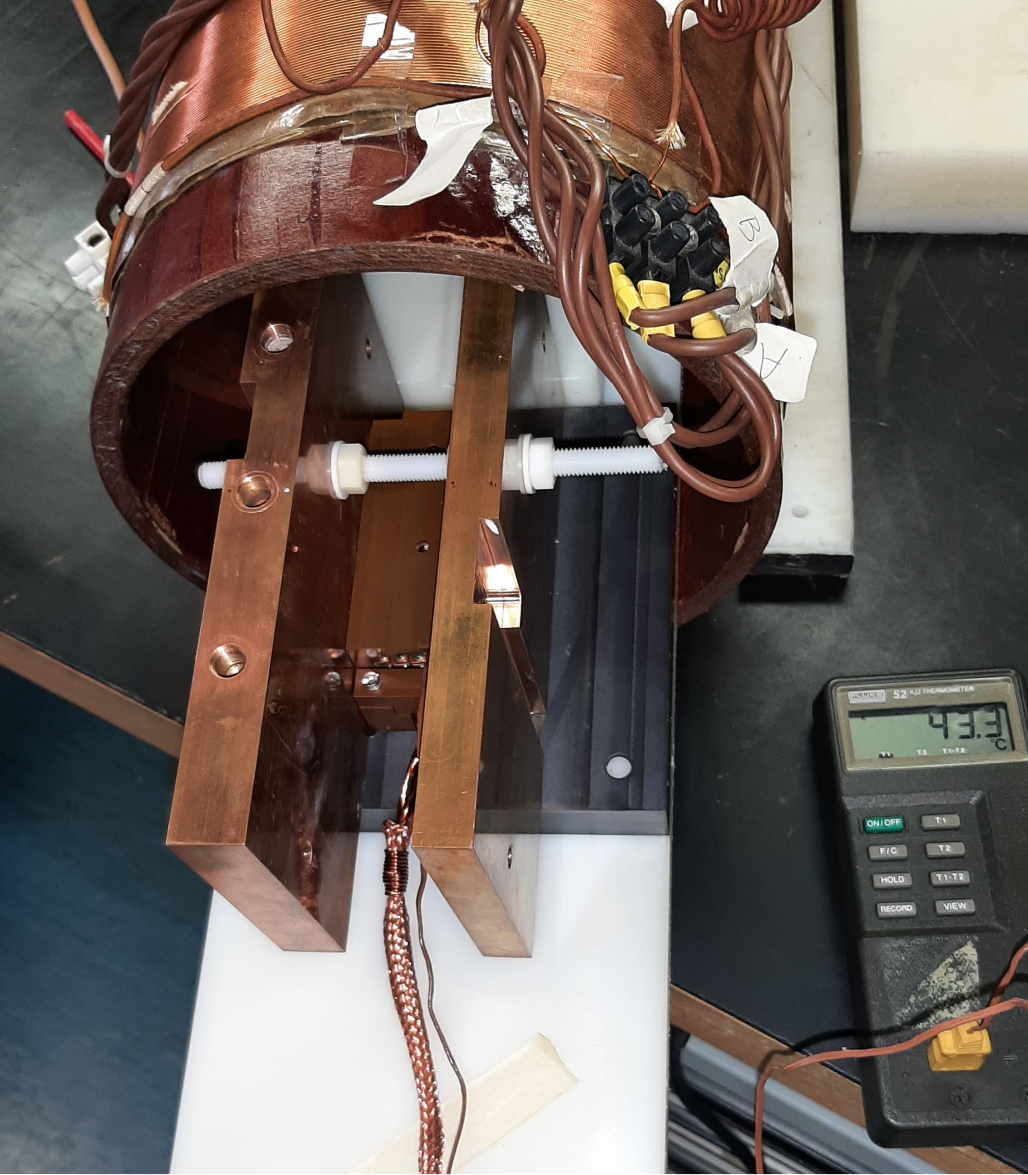
The operation of the sensing part was tested at an increasing temperature of up to 44 °C, the temperature was stabilized by inserting the sensing part between a pair of thick copper blocks.

A DC current was applied to the solenoid, so as to produce an induction field up to 10 mT in the centre of the solenoid, where the sensing part was placed. The current in the solenoid as well as the output signal of the fluxgate sensor were measured using HP-Agilent 34401A multimeters. A linearity test at room temperature and with the original 10 m long connection cable was carried out as reference. Then, the same test was repeated with the sensing part assembled into a sandwich between two heated copper plates, as shown in Figure 12. A thermocouple was inserted inside the containing box in order to monitor when a temperature of 44 °C was reached on the sensing part.

**Figure 13:**
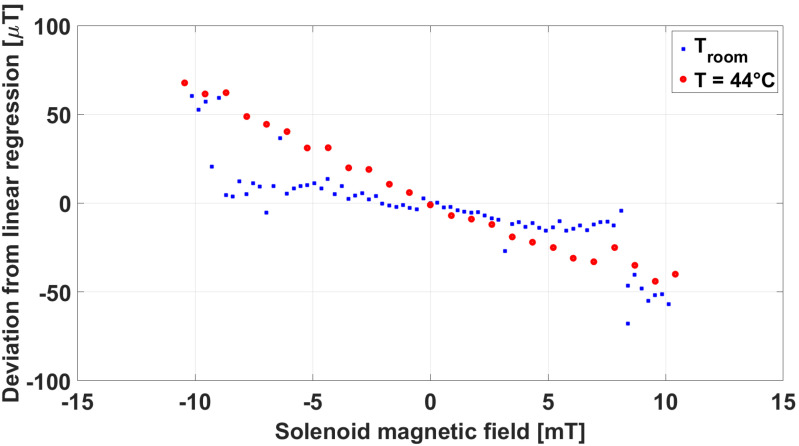
Comparison of linearity tests carried out at Consorzio RFX at room temperature and at 44 °C.

In Figure 13, the comparison between the linearity error at room temperature and at 44 °C is reported. In both conditions, the error exceeded the requirement of ≤20 μT indicated in Table 1, but showed a similar behaviour both at room temperature and at 44 °C. We ascribed this behaviour to the presence of a large ferromagnetic structure not far from the area where this test was carried out at Consorzio RFX. As shown in Table 1, the absolute accuracy of the sensor (10 μT) was guaranteed within the measurement range of 2 mT. The extension of the measurement range to 10 mT was obtained by means of an internal feedback compensation of the sensor. It is likely that, when the field measured exceeded 4–5 mT, this compensation was less effective at higher temperature. On this basis, this comparison can definitely be meaningful of the negligible impact of the temperature of the sensing part on the magnetic field measurement.

**Figure 14:**
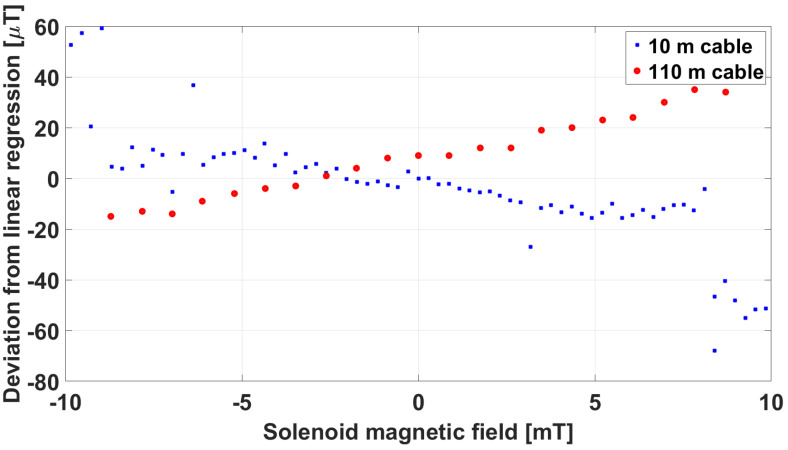
Comparison of linearity error carried out at Consorzio RFX at room temperature with the original 10 m cable and a 100 m test cable.

A commercial multipolar cable, with twisted and shielded pairs of 1 mm^2^ cross-section conductors, 100 m long, was adopted as an extension cable to test the capability of the front-end electronics to correctly operate at a much larger distance from the sensing part. In Figure 14, the comparison between the linearity error with the original 10 m connection cable and with the additional 100 m extension cable is reported.

Here, we could see an opposite sign of the linearity error, but of similar value to the original cable. The sensor was therefore considered to be capable of operating under realistic ITER conditions. The capability of the front-end electronics to work properly with a DC input voltage lower than the nominal 12 V was also verified. Finally, an insulation test was carried out to verify the voltage-holding capability of the conductors inside the sensing part with respect to ground. To this purpose, the sensing part was wrapped with a metallic sheet and a voltage was applied between the connection cable terminals and the metallic sheet, using a Yokogawa MY600 digital insulation tester (megaohmeter). A voltage of up to 1 kV was applied in sequence, confirming an insulation resistance greater than 5 MΩ.

### 4.3. Field Tests at Neutral Beam Test Facility

Further tests will be carried out inside the MITICA vacuum vessel, when it is available (possibly at the end of 2023), in order to verify the correct operation in real vacuum conditions. An external magnetic source will be adopted to produce a known field distribution in the region where the sensors will be placed. The stability of the measurement in time, together with the temperature inside the containing box will be monitored. Depending on the results of the planned vacuum tests and temperature measurements, it may be possible to reduce the size of the sensing part. This will considerably reduce the production costs of sensors and increase the possible applications of these sensors in other parts of the ITER experimental device.

## 5. Discussion and Conclusions

Magnetic field sensor prototypes, suitable for measuring a stationary and slow-varying magnetic field of up to 10 mT in the ITER vacuum and nuclear environment, were developed and realized. The fluxgate technology was chosen in view of the possibility to sense a stationary field with a high accuracy and stability. The major challenges were related to the use of vacuum-compatible and radiation-hard materials and to the extension of the usually limited measurement range of fluxgate sensors. The heating of the sensing part also had to be reduced in view of the need to operate in vacuum. The specially developed Vacquier-type fluxgate sensor working in compensation mode allowed us to fulfil all requirements and to fit the various constraints. Several tests were successfully performed, both at the Stefan Mayer Instruments GmbH factory and in house at Consorzio RFX, and reported, showing the complete compliance with all requirements.

The results of the sensor tests also demonstrated that the integrated physics and engineering design effort, applied in the definition of the magnetic sensor specifications (reported in Table 1), allowed the realization of a new class of magnetic sensors, necessary for the operability of the ITER experimental device.

The use of the fluxgate sensor described in this paper is not limited to nuclear fusion reactor applications. The special properties of this fluxgate sensor (extended measurement range, vacuum compatibility, and radiation hardness) make it also useful for other applications. The extended measurement range is for example useful for active shielding of the ramping field of NMR magnets. Vacuum compatibility enables the sensor installation inside the vacuum chambers of devices using charged particle beams such as electron microscopes, e-beam lithography systems or ion-beam spectrometers. This makes it possible to install the sensor of an active magnetic field compensation system closer to the charged-particle beam resulting in a better beam stability in nonhomogeneous field conditions.

Future developments may also include versions where one or more of the special properties are replaced by other features. For example, a sensor with a 10 mT measurement range, but a higher bandwidth and resolution than the present prototype can be very useful for powerline AC magnetic field active shielding of devices which produce a large DC magnetic field offset such as MRI scanners. For this purpose, the sensor excitation frequency could be increased while relaxing the power consumption limit for vacuum use.

## Figures and Tables

**Table 1:** Summary of sensor measurement specifications. The ones confirmed by the acceptance tests are highlighted in **bold**.

Par.#	Parameter	Value
1	measurement range	**±2.0 mT** (extended range ±10 mT)
1	Measurement range	**±2.0 mT** (extended range ±10 mT)
2	Measurement sensitivity	**≤10** μ **T**
3	Absolute accuracy	**≤20** μ **T**
4	Analogue output voltage range	**±10 V**
5	Offset due to temperature variations	<500 nT/K
6	Operating temperature range	20–40 °C
7	Time derivative of magnetic field (max)	**10 mT/s**
8	Measurement bandwidth	**DC—10.0 Hz (−3 dB)**
9	Withstand voltage between sensor cables and ground (max)	1 kV for 60 s
10	Alignment of sensor package with sensor reference surfaces	**±0.5**°
11	Immunity to transverse magnetic field	**<−40 dB**

## Data Availability

Not applicable.
